# Recent Advances in Nano-Therapeutic Strategies for Osteoarthritis

**DOI:** 10.3389/fphar.2022.924387

**Published:** 2022-06-21

**Authors:** Xinjing Guo, Jia Lou, Fazhan Wang, Daoyang Fan, Zhihai Qin

**Affiliations:** ^1^ Medical Research Center, The First Affiliated Hospital of Zhengzhou University, Zhengzhou University, Zhengzhou, China; ^2^ Department of Orthopedic, The First Affiliated Hospital of Zhengzhou University, Zhengzhou University, Zhengzhou, China; ^3^ Academy of Medical Sciences, Zhengzhou University, Zhengzhou, China

**Keywords:** nanotechnology, nanoparticle, osteoarthritis, nanotherapeutic strategies, nanomaterials

## Abstract

Osteoarthritis (OA) is the most common type of arthritis and the leading cause of disability globally. It tends to occur in middle age or due to an injury or obesity. OA occurs with the onset of symptoms, including joint swelling, joint effusion, and limited movement at a late stage of the disease, which leads to teratogenesis and loss of joint function. During the pathogenesis of this degenerative joint lesion, several local inflammatory responses are activated, resulting in synovial proliferation and pannus formation that facilitates the destruction of the bone and the articular cartilage. The commonly used drugs for the clinical diagnosis and treatment of OA have limitations such as low bioavailability, short half-life, poor targeting, and high systemic toxicity. With the application of nanomaterials and intelligent nanomedicines, novel nanotherapeutic strategies have shown more specific targeting, prolonged half-life, refined bioavailability, and reduced systemic toxicity, compared to the existing medications. In this review, we summarized the recent advancements in new nanotherapeutic strategies for OA and provided suggestions for improving the treatment of OA.

## 1 Introduction

Arthritis is a clinically common and frequently-occurring disease. According to its etiology, clinical manifestations, biochemical tests, and genetic examinations, arthritis can be divided into several types; however, osteoarthritis (OA) is clinically the most common type. The incidence of knee OA is 33% in the population aged 60–70 and about 43.7% in the population over the age of 80. It is the main bone disease causing disability (Shane and Loeser, 2010). Pathological features of OA mainly include the degradation of the cartilage extracellular matrix (ECM) components, synovial inflammation, osteophyte formation, joint space narrowing, ligament and meniscus changes, subchondral bone remodeling, and the loss of joint functions (Yuan et al., 2020; Peng et al., 2021). Patients with advanced OA rely on joint replacement surgery to restore joint functions. This causes a huge social and economic burden and impairs the quality of life of the patients (Hiligsmann et al., 2013). Osteoarthritis (OA) most commonly affects the knee joints, and the next most commonly affected sites are the hands and hips. No specific treatment can reduce the progression of hand OA (Favero et al., 2022).

The progression of OA is complex with the involvement of the whole joint, which is involved and driven by a variety of inflammatory cytokines, including cytokines, reactive oxygen species and nitrogen species, and matrix metalloproteinases. The disease alters the articular cartilage, meniscus, synovial membrane, subchondral bone, ligaments, capsule, and periarticular musculature (Loeser et al., 2012). It also has a strong hereditary background (Ratneswaran and Kapoor, 2021). Variant alleles of several genes are often highly expressed in the cartilage, which increases the risk of developing OA (Richard et al., 2020). Additionally, the risk factors of OA also differ between sexes (Mchugh, 2021). During the development of primary osteoarthritis caused by aging and genetics and secondary osteoarthritis induced by trauma and obesity, fibrillation appears on the cartilage surface, with gradual degradation of the collagen II network and glycosaminoglycans (Mort and Billington, 2001). OA is also associated with a variable degree of synovial inflammation that can be induced by the damage and dysfunction of the cartilage (Scanzello, 2017). During the progression of OA, macrophages, synovial fibroblasts, and chondrocytes produce cytokines, chemokines, proteases, and reactive oxygen species (ROS) (van den Bosch, 2021; Wang et al., 2021). Cytokines such as interleukin-1 (IL-1) and tumor necrosis factor-*α* (TNF-*α*) promote synovitis, inhibit chondrocyte viability, and induce the production of many proteases. The degeneration and remodeling of different tissues in the joint highly depend on the activity of different proteases, such as matrix metalloproteinase (MMP) (Xia et al., 2014; Grassel et al., 2021), which can degrade the cartilage matrix. Other growth factors are also involved in the progression of OA, such as transforming growth factor *β* (TGF-*β*), fibroblast growth factor (FGF)-2, and FGF-18 (Fortier et al., 2011). The related signaling pathways in the progression of OA might be therapeutic targets.

Besides physiotherapy and exercise, available drug therapies for OA, such as non-steroidal anti-inflammatory drugs (NSAIDs), opioids, and glucocorticoids, are the main ways to treat OA. However, the long-term systemic administration of drugs can cause severe side effects, such as gastrointestinal complications, an increase in cardiovascular risk, and osteoporosis, which are well-known limitations (Da et al., 2017). Most clinical therapy mainly targets pain reduction, functional improvement, and artificial joint replacement. Some molecular therapies, such as the injection of the hyaluronic acid polymer in the intra-articular cavity, are also widely used in osteoarthritis treatment for lubricating joints and promoting bone repair. This delays the development of arthritis and improves joint mobility but does not reverse the progression of OA (Kavanaugh et al., 2016; Maudens et al., 2018a). Besides joint replacement surgery, there is no effective treatment to decelerate the progression of OA or retard the irreversible degradation of cartilage. Therefore, the development of novel drugs and therapeutic strategies is necessary for more effective OA treatment.

**FIGURE 1:**
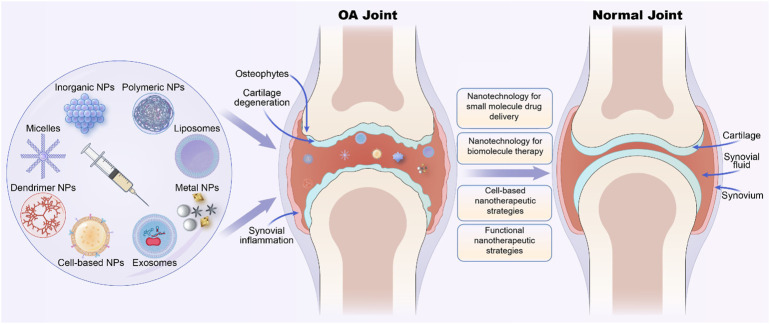
Various nano-therapeutic strategies based on different nanoparticles for osteoarthritis (OA) treatment.

Nanotechnology is an interdisciplinary field of science for studying and manipulating nanoparticles that are usually between 1 and 100 nm. Nanoparticles (NPs) often have unique properties due to their scale structure, such as size effects, interfacial phenomena, and quantum effects, and thus, they have multiple advantages and novel functions (Jeevanandam et al., 2018). Numerous nanoparticle-based drug delivery systems such as polymer, micelles, liposomes, dendrimers, polymer nanoparticles (PNPs), and inorganic NPs have been investigated for intravenous and intra-articular (IA) injection therapy of OA. These nanotherapeutic strategies not only improve drug targeting and efficient drug delivery but also improve drug solubility and stability. Additionally, they prevent drug dispersion and degradation in body fluids and prolong drug circulation and retention time, thus improving drug efficacy and reducing adverse drug reactions (Rothenfluh et al., 2008; Gu et al., 2013). Many works of nanomaterials in OA treatment have been excellently summarized (Bottini et al., 2016; Cheng et al., 2017). However, nanotechnology and materials are developing rapidly, and the functions to meet different needs are constantly improving. Various biomimetic camouflage strategies have also been developed and studied in the treatment of various diseases including OA. Cell membranes derived from erythrocytes, platelets, and tumor cells have been used for camouflage and modification of nanoparticles. These nanocarriers can obtain more advantages in drug delivery and surface function after being coated with biomembranes (Zhang et al., 2021). Thus, in this review, we discussed the recent advances of OA-related novel nanotherapeutic strategies based on different NPs (Figure 1) and mainly focused on the following aspects to summarize, including nanotechnologies for small molecule drug delivery, nanoparticles for biomacromolecule therapy, cell-based nanotherapeutic strategies, and other functional nanomaterials that attracting more and more attention.

## 2 Novel Nanotherapeutic Strategies for OA Therapy

### 2.1 Nanotechnology for Small Molecule Drug Delivery in OA Treatment

Short biological half-lives significantly limit the application of numerous pharmacologically active small-molecule drugs. Studies have demonstrated the beneficial properties of nanocarriers for targeted drug delivery and sustained release, which makes them an effective tool for enhancing the efficacy of multiple drugs or helping in the diagnosis of OA. Liu et al. reported the synthesis and activity of six targeted polymeric (PEG-b-PLA) nanoparticles for OA treatment as adenosine receptor agonists (Liu et al., 2019), which suggested that the combination of adenosine with biodegradable nanoparticles can significantly extended the therapeutic effects. The oral administration of NSAIDs (such as diclofenac, DIC) and the intra-articular injection of glucocorticoids (such as dexamethasone, DEX) are the common therapeutic strategies in OA (Nakata et al., 2018). However, both drugs show severe adverse effects, including the risk of toxicity (Roth and Fuller, 2011).

Liposomal nanoparticles can optimize the therapeutic and delivery efficiency of various formulations and have been used clinically for several decades (Eldem and Eldem, 2018). Chang et al. designed a novel OA treatment formulation, hyaluronic acid (HA)-Liposomal (Lipo)-DIC/DEX, to combat joint pain. The nanoparticles were formulated by constructing DIC with DEX-loaded nanostructured lipid carriers Lipo-DIC/DEX mixed with hyaluronic acid (HA) for prolonged OA treatment (Chang et al., 2021). The study suggested that HA-Lipo-DIC/DEX might be a promising system for osteoarthritis pain control as it reached the effective working concentration in 4 h and sustained the drug-releasing time for at least 168 h with no significant toxicities. The intra-articular injection of HA-Lipo-DIC/DEX considerably inhibited knee joint inflammation over 4 weeks. The injection of a single dose could also decrease the inflammation to 77.5 ± 5.1% from the initial level of inflammation over that duration. Rapamycin might be administered for treating osteoarthritis (OA) (Matsuzaki et al., 2014; Pal et al., 2015). Low-intensity pulsed ultrasound (LIPUS) also exhibits anti-OA effects (Ji et al., 2015; He et al., 2018). Chen et al. combined liposome-encapsulated rapamycin (L-Rapa) with LIPUS to promote the anti-osteoarthritic effects of rapamycin. This nanotherapeutic strategy had the most consistent and effective anabolic and anti-catabolic effects on human OA chondrocytes (HOACs) and spontaneous OA guinea pigs. These results indicated that IA delivery of liposome-encapsulated rapamycin has significant anti-inflammatory effects on spontaneous OA guinea pigs (Chen et al., 2020).

Among the potent inflammatory mediators involved in the progression of OA is the family of secreted phospholipase A2 (sPLA2) enzymes, a heterogeneous group of enzymes that can specifically recognize and catalytically hydrolyze the sn-2 ester bond of glycerophospholipids, releasing free fatty acids such as arachidonic acid (AA) and lysophospholipids (Burke and Dennis, 2009). Wei et al. found that the concentration of the sPLA2 enzyme increases in the articular cartilage in human and mouse OA cartilage tissues, and the inhibition of sPLA2 activity might be an effective pharmacological treatment strategy for OA (Wei et al., 2021). They developed an sPLA2-responsive and nanoparticle (NP)-based intervention platform for OA management by incorporating an sPLA2 inhibitor (sPLA2i) into the phospholipid membrane of micelles. The engineered sPLA2i-loaded micellar NPs (sPLA2i-NPs) penetrated the cartilage matrix, inhibited sPLA2 activity strongly, suppressed the inflammatory signals, and mitigated the progression of OA following direct delivery into knee joints. This indicated that sPLA2i-NPs can be a promising therapeutic tool for the treatment of OA.

**FIGURE 2:**
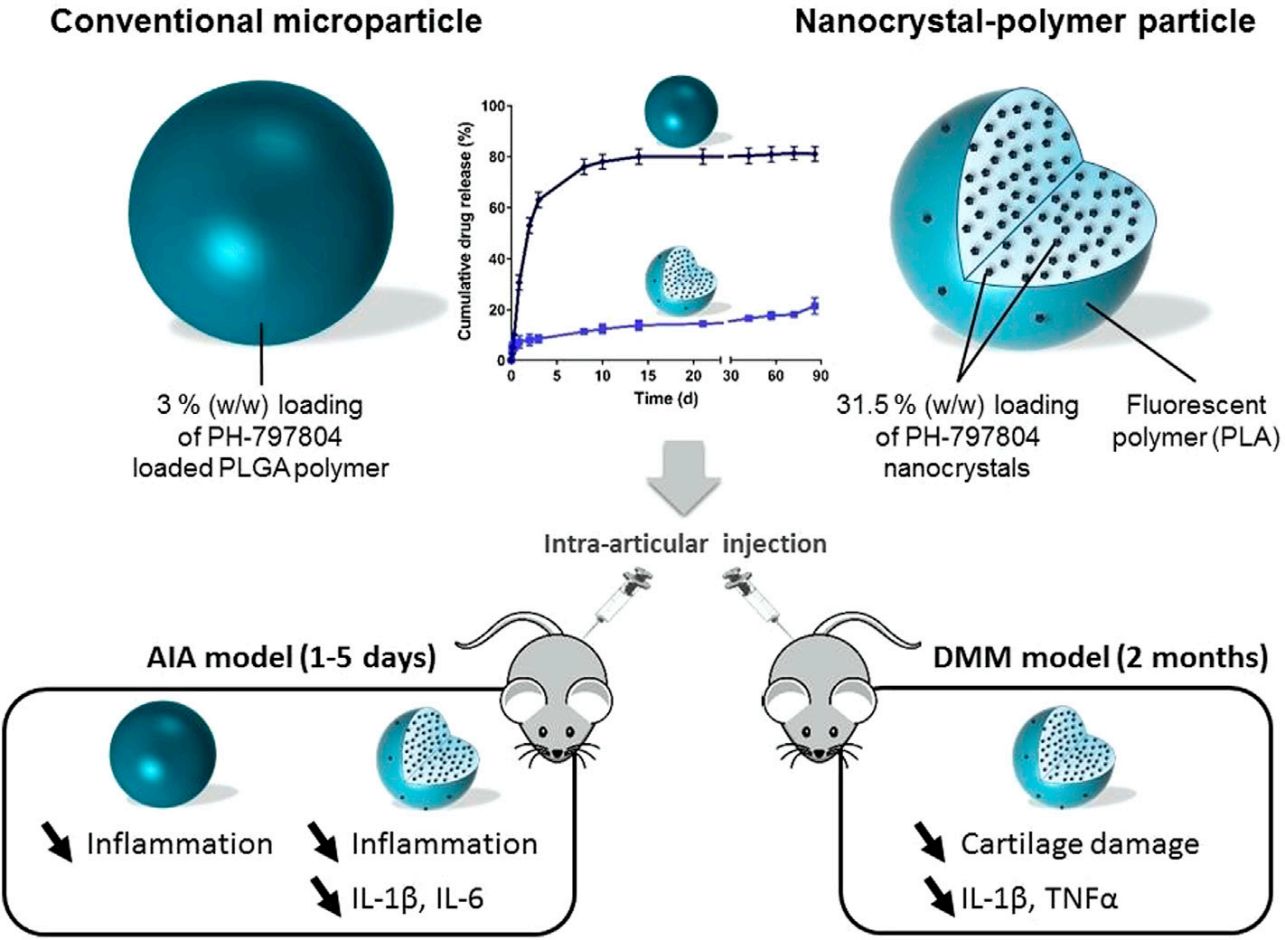
Drawing of the PH-797804 (PH-NPPs) with extendedrelease properties over several months compared to conventional PH microparticles for intra-articular treatment inflammatory and mechanistic murine models. Reproduced with permission (Maudens et al., 2018c). Copyright 2018, Elsevier.

Furthermore, the challenges of drug delivery to the cartilage can be overcome by using cationic nanoparticles less than 10 nm in diameter (e.g., Avidin) that have ideal characteristics for targeted intra-cartilage drug delivery (Bajpayee et al., 2014). Bajpayee et al. proposed a nanotherapeutic strategy that conjugated Avidin with dexamethasone (DEX) using fast (ester) and slow, pH-sensitive release (hydrazone) linkers. DEX was rapidly released from the Avidin-delivered DEX with high bioactivity, and a single dose of Avidin-DEX significantly inhibited the cytokine-induced loss of sulfated-glycosaminoglycan (sGAG), suppressed IL-1*α*-induced cell death, and enhanced the sGAG synthesis level *in vitro* (Bajpayee et al., 2016). Signaling through the p38*α*/*β* mitogen-activated protein kinase (MAPK) plays an important role in OA. PH-797804 (PH) is a potent and selective inhibitor of p38*α*/*β* MAPK but failed to demonstrate clinical results (Haller et al., 2020), suggesting that PH requires a delivery system with sustained/extended release that can last for months. Maudens et al. proposed (PH-797804)-loaded nanostructures (PH-NPPs) for the management of OA (Maudens et al., 2018c). PH-NPPS were prepared by wet milling and stabilized with D-ɑ-tocopheryl polyethylene glycol 1,000 succinate and then embedded into fluorescent particles (Figure 2). The PH-NPPs were large (diameter: 14.2 µm), showed high drug loading (31.5%), effectively prolonged drug release, and exhibited good biocompatibility. Additionally, PH-NPPs remained in the joint and adjacent tissues for 2 months and decreased the levels of inflammatory factors, including IL-1*β*, IL-6, and IL-17, which inhibited inflammation and joint damage.

### 2.2 Nanotechnology for Biomacromolecule Therapy in OA Treatment

Different from small-molecule therapeutics, peptides and biologics provide unique opportunities to modulate particular intracellular targets, with high selectivity, high potency, predictable behavior, and limited side effects. Biologics, such as therapeutic peptides and RNA-based formulations for gene therapy, were investigated for regulating their intracellular targets, but had a low clinical impact (Skalko-Basnet, 2014; Sharfstein, 2018). However, these intracellularly acting peptides, proteins, and nucleic acids are often less effective in therapeutics as they cannot penetrate the cellular and endolysosomal membranes effectively. Some delivery systems lack potency due to low uptake and/or entrapment and degradation in endolysosomal compartments (Evans et al., 2019). As summarized in Table 1, novel nanocarriers and nanotechnologies for biomacromolecule therapy are promising in OA treatment.

**TABLE 1:** Nanotechnology for biomacromolecule therapy in OA treatment.

Table	Cargo	Composition	Model	Animal/Delivery route	Outcome	Ref.
Nanotechnology for gene therapy	MMP13 siRNA.	MMP13 siRNA, mAbCII, PEG and DB	PTOA induced by noninvasive repetitive joint loading	Mice/i.a.	MMP-13 ↓, protected against meniscal mineralization and osteophyte formation	Bedingfield et al. (2021a)
	siRNA (Postn)	Peptide-nucleotide polyplex and sirna (Postn)	OA induced by DMM.	Mice/i.a.	Subchondral bone sclerosis, BV/TV, vBMD, and heterotopic ossification, MMP-13, ADAMTS-4 ↓	Duan et al. (2021)
	miR-140	G5-AHP and miR-140	OA induced by DMM.	Mice/i.a.	Col2 ↑, MMP, ADAMTS5 ↓	Li et al. (2022)
Other NP-based biomacromolec-ule therapy	IGF-1	IGF-1; PEGylated PAMAM dendrimer cationic nanocarrier	OA induced by ACLT + MMx	Rats/i.a.	Degenerated cartilage area, degenerated surface cartilage width, total osteophyte volume ↓	Li et al. (2015)
	HAS2	Biodegradable mesoporous silica nanoparticles and HA.	OA induced by injecting CFA.	Rats/i.a.	Improved HA retention rate, IL-1*β*, TNF*α* ↓	Li et al. (2019)

Abbreviations: ACLT, anterior cruciate ligament transection; ADAMTS, a disintegrin and metalloproteinase with thrombospondin motifs; CFA, complete Freund’s adjuvant; Col2: type II collagen; DB, DMAEMA (2-(dimethylamino)ethyl methacrylate)-co-BMA (butyl methacrylate); DMM, Destabilized medial meniscus; G5-AHP, generation 5 polyamidoamine; HA, hyaluronic acid; HAS2, hyaluronan synthase type 2; IGF, insulin-like growth factor; IL-1*β*, interleukin 1*β;* mAbCII, Col2 monoclonal antibody; MIA, Monosodium iodoacetate; miR-140, microRNA-140; MMP13, matrix metalloproteinases; MMx, medial meniscectomy; Postn, Periostin; PAMAM, polyamidoamine; PEG, poly (ethylene glycol); PTOA, Post-traumatic OA; siNPs, siRNA nanoparticle complexes.

#### 2.2.1 Nanotechnology for Gene Therapy

Gene therapy might be used for the treatment of human diseases, including cancer, hereditary diseases, and other disorders, through the delivery of therapeutic nucleic acids (DNA or RNA) to targeted tissues or cells (Kaufmann et al., 2013). However, gene therapy has major challenges, mainly related to the improvement of the delivery efficiency and the *in vivo* stability of functional genes or nucleic acids, to prolong the effective functional time of gene carriers and perform on-demand treatment of diseases by microenvironmental responses. Therefore, multiple local delivery approaches have been applied to improve the distribution of therapeutic genes in target tissues (Emanueli et al., 2001) and also promote non-viral gene delivery using nanocarriers. Additionally, scaffolds might be an excellent approach for disease therapy, including OA treatment (Gonzalez Fernandez et al., 2018). Some studies have shown the role of periostin in OA. The expression of periostin aggravates the damage of articular cartilage after a knee injury. Duan et al. found that the expression of periostin in mice with post-traumatic OA was significantly inhibited using intra-articular (IA) delivery of a peptide-siRNA nanoplatform. Moreover, the expression of matrix metalloproteinase 13 (MMP-13), subchondral bone sclerosis, bone volume/total volume, volumetric bone mineral density, and heterotopic ossification were also significantly lower in treated mice. The Osteoarthritis Research Society International (OARSI) cartilage damage score was also considerably lower in mice that were administered periostin siRNA (Duan et al., 2021). The administration of the periostin-siRNA nanocomplex is a promising approach to alleviating the severity of joint degeneration in OA.

**FIGURE 3:**
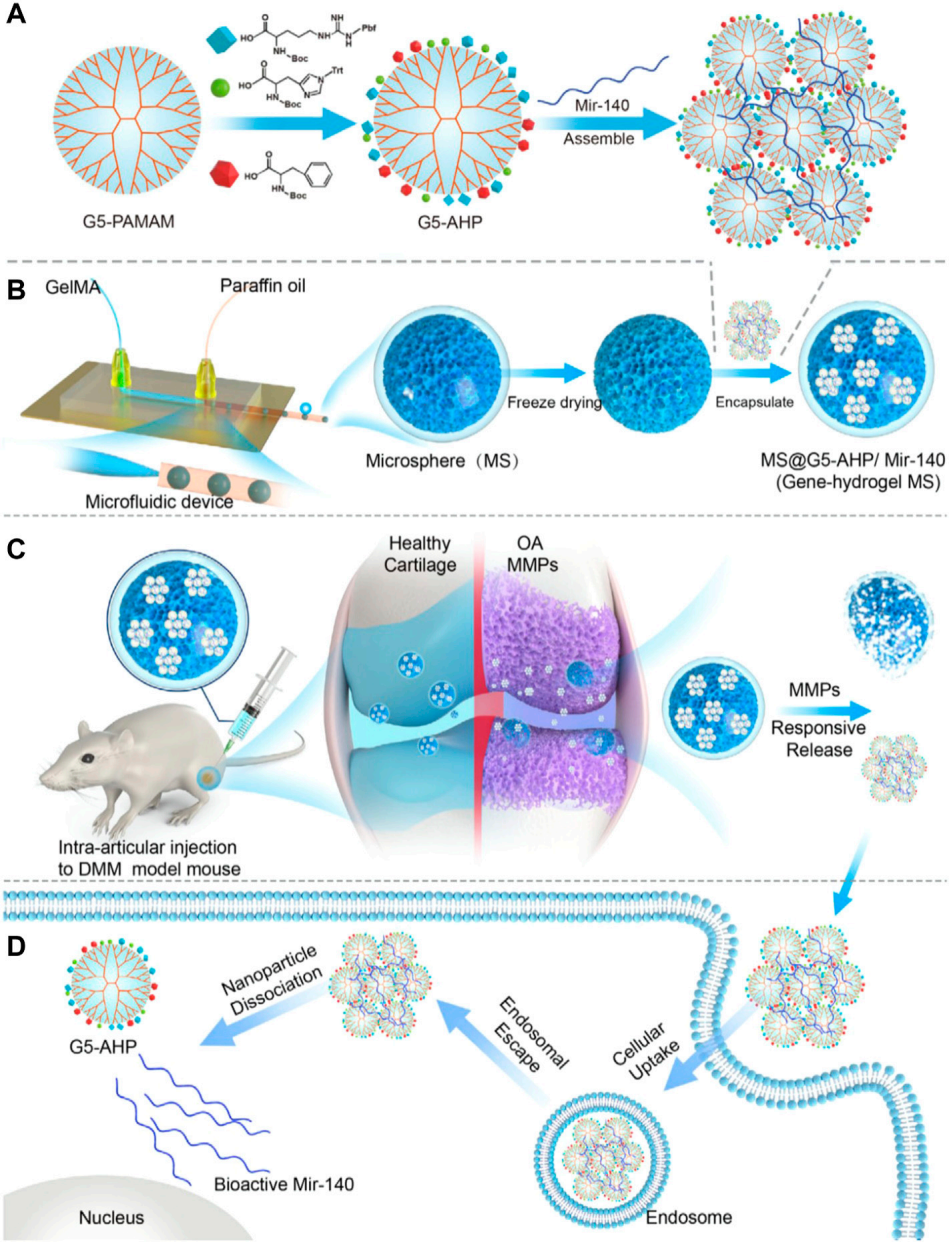
Gene-hydrogel microspheres for the treatment of OA. **(A)** preparation of G5-AHP and G5-AHP/miR-140. **(B)** Gene-hydrogel MSs. **(C)** Injection with MS@G5-AHP/miR-140 into the articular space to alleviate the progression of OA. **(D)** Endocytosis of G5-AHP/miR-140 polyplexes and the release of miR-140. Reproduced with permission (Li B, 2022). Copyright 2022, Nature Publishing Group.

Bedingfield et al. locally injected nanoparticles modified with antibodies targeting type II collagen and carrying small interfering RNA targeting MMP-13, they reduced the expression of MMP-13 and protected the integrity of the cartilage and the joint structure (Bedingfield et al., 2021b). The researchers also designed small interfering RNA (siRNA)-loaded nanoparticles (siNPs) that were encapsulated in shape-defined poly(lactic-co-glycolic acid) (PLGA)-based microplates to maintain siNPs in the joint significantly longer than delivery of free siNPs. They enabled long-lasting therapy for post-traumatic OA by silencing MMP13 (Bedingfield et al., 2021a). Li et al. constructed a responsive MS@G5-AHP/miR-140 “nano-micron” combined microfluidic gene-hydrogel microspheres (MSs) to alleviate the degradation of articular cartilage as a novel strategy for regional gene therapy (Li et al., 2022). They used a multifunctional gene vector, arginine, histidine, and phenylalanine-modified generation 5 polyamidoamine (G5-AHP) to prepare G5-AHP/miR-140 nanoparticles and then trapped them in GelMA MSs (Figure 3). The system not only increased the cellular uptake efficiency of the therapeutic microRNA-140 and the transfection efficiency but also extended the effective functional time of microRNA-140 after injection and facilitated the on-demand response of the disease microenvironment. Chen et al. constructed photothermal-triggered nitric oxide (NO) nanogenerators NO-Hb@siRNA@PLGA-PEG (NHsPP). NHsPP, combined with siRNA, could efficiently convert absorbed NIR light energy into sufficient heat to trigger NO production. The platform demonstrated controlled NO release in RAW 264.7 cells and live OA mice. It efficiently treated OA by inhibiting macrophage inflammation and effectively prevented cartilage erosion (Chen et al., 2019).

#### 2.2.2 Other NP-Based Biomacromolecule Therapy

For the efficient treatment of osteoarthritis (OA), the local release of a high therapeutic dose over a long time is advantageous. Several biomacromolecules, such as anabolic growth factors, have been delivered to target cells inefficiently and inadequately in clinical trials. Insulin-like growth factor 1 (IGF-1) is an anabolic growth factor that promotes chondrocyte survival, proliferation, and biosynthesis of cartilage matrix macromolecules (Schmidt et al., 2006; Li et al., 2015). It also shows anti-inflammatory effects in cartilage tissue. IGF-1 might be more effective in the treatment of OA if delivered directly to chondrocytes residing deep within dense anionic cartilage tissues. Geiger et al. conjugated IGF-1 to a cationic nanocarrier and performed direct intra-articular injection for targeted delivery to chondrocytes and retention within the joint cartilage. Treatment of OA rats with dendrimer-IGF-1 reduced the extent of cartilage degeneration by 60% and volumetric osteophyte burden by 80%, relative to the extent of the damage in untreated OA rats. Additionally, dendrimer-IGF-1 rescued cartilages and bones more effectively than free IGF-1, which indicated that conjugation to a cartilage-penetrating PEGylated dendrimer can improve the delivery and efficacy of disease-modifying biologic drugs in the treatment of OA (Geiger et al., 2018).

**FIGURE 4:**
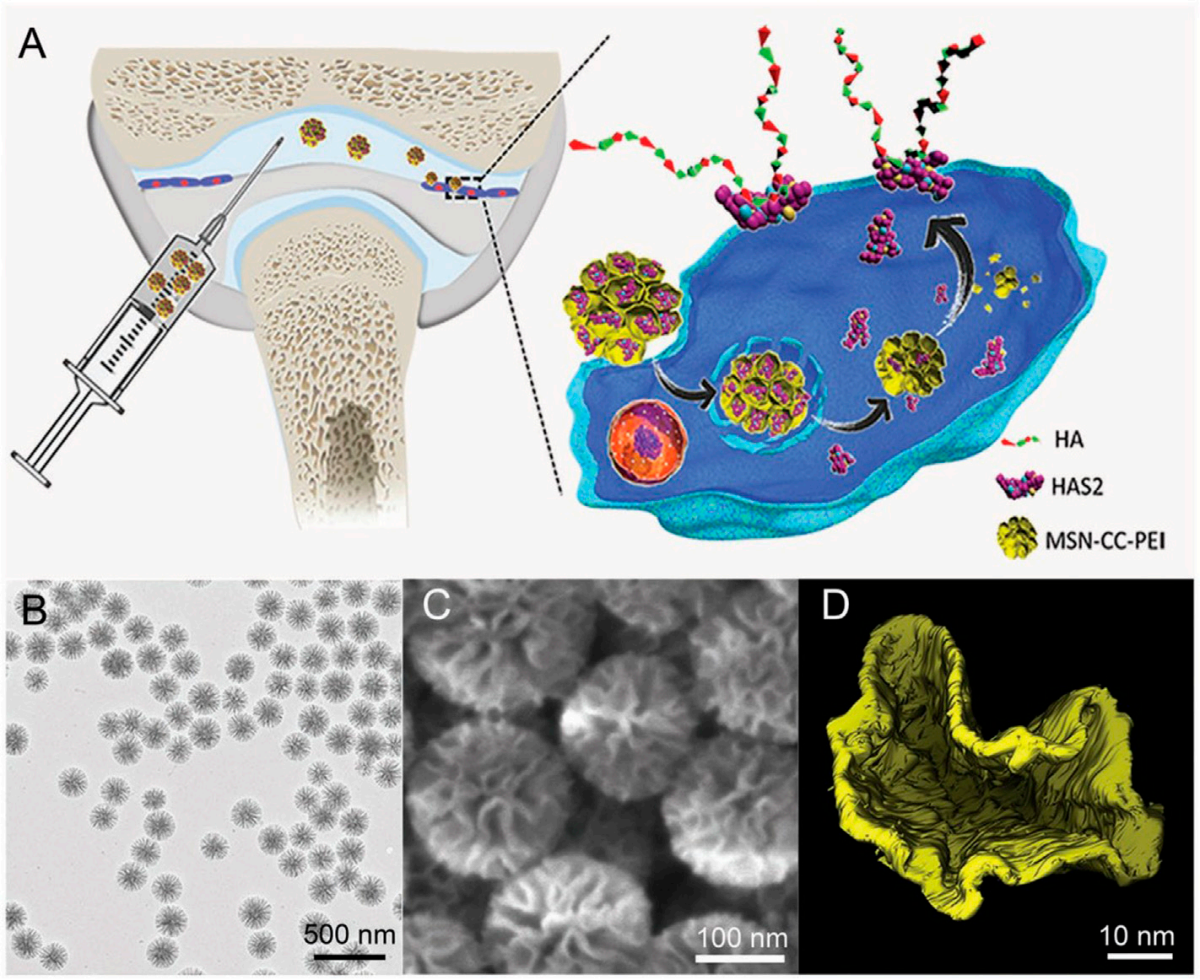
Illustration of the cellular delivery of HAS2 to synoviocytes using nanoparticles **(A)**. Representative image of the nanoparticles examined with TEM **(B)**, SEM **(C)**, and the reconstructed pore structure by electron tomography (ET) technique **(D)**. Reproduced with permission (Li et al., 2019). Copyright 2019, Wiley-Blackwell.

External hyaluronic acid (HA) supplementation is a common treatment for osteoarthritis but requires multiple injections due to its generally rapid degradation. Additionally, low molecular weight hyaluronic acid can cause bone destruction. Treatment of OA by promoting endogenous macromolecular hyaluronic acid in the joint has some advantages, and thus, the therapeutic strategy to increase the level of hyaluronic acid synthetic enzyme II (HAS2) in the synovial cells is necessary. Mesoporous silica nanoparticle (MSN) is regarded as an ideal protein drug carrier due to its advantages of high protein loading, good biocompatibility, and easy surface modification with organic groups (Wu et al., 2013; Li et al., 2014; Watermann and Brieger, 2017). Li et al. showed that biodegradable mesoporous silica nanoparticles successfully delivered hyaluronan synthase type 2 (HAS2) into synoviocytes from the temporomandibular joint (TMJ) and generated endogenous HA with high molecular weights (Figure 4). This strategy promoted endogenous HA production and inhibited the synovial inflammation of OA *in vitro* and *in vivo*. Such nanotherapeutic techniques also repaired bone defects and maintained normal morphology in another rat OA bone defect model (Li et al., 2019). The delivery of interleukin-1 receptor antagonist (IL-1Ra), a natural protein inhibitor of IL-1 that can modulate IL-1-based inflammation, emerged as a promising approach for osteoarthritis treatment (Chevalier et al., 2005; Chevalier et al., 2009). However, these anti-inflammatory therapies were limited due to the lack of effective and suitable drug delivery materials, which caused rapid clearance and reduced potency of the therapeutic substances over time. Whitmire et al. designed a system consisting of a new block of copolymer that self-assembled into nanoparticles and efficiently incorporated the IL-1Ra protein onto their surface for the intra-articular delivery of anti-inflammatory proteins. The IL-1Ra nanoparticles maintained higher protein bioactivity *in vitro*, bound specifically to synoviocyte cells more efficiently, and inhibited signaling mediated by IL-1 more strongly compared to soluble IL-1Ra. The IL-1Ra tethered nanoparticles significantly increased IL-1Ra retention in rat knee joints and prolonged IL-1Ra half-life without inducing degenerative changes in the structure and composition of the cartilage (Whitmire et al., 2012).

### 2.3 Cell-Based Nanotherapeutic Strategies for OA

The incorporation and modification of membrane proteins enables nanoparticles to effectively avoid being cleared by the monocyte phagocytic system, better pass through biological barriers or reach target tissues, such as tumors or sites of inflammation, and can further exert a variety of biological functions (Zhang et al., 2021). Various biomimetic cell-based nanoparticles are also emerging in the treatment of OA. As shown in Table 2, some recent studies have demonstrated the advantages of novel cell-based nanotherapeutic strategies.

**TABLE 2:** Cell-based nanotherapeutic strategies for OA.

Source (cell)	Composition	Disease model	Animal/Delivery route	Outcome	Ref.
MSCs	NGs: MSCs’ cytoplasmic-membrane-based nanoparticles	OA induced by a surgical destabilization of the knee medial meniscus (DMM)	Mice/i.a.	PGE2, IL-6, IL-8, COX2, ADAMTS5, MMP13 ↓, ACAN ↑	D'Atri, et al., 2021
M2 macrophages	Macro-phage membrane and inflammation-responsive nanogel	OA induced by papain injection	Mice/i.a.	Promoted pro-liferation of chondrocytes, IL-1*β*, TNF-*α*, IL-6, IL-17 ↓	Ma et al. (2021)
M2 macrophages	M2 macrophage membrane, gold nanoparticles	OA induced by P2 chondrocytes stimulated with IL-1*β*	Rats/—	MMP13, NO, CCL5, IL6, IL8 ↓, sGAG, ACAN, COL2A1, COL6A1 ↑	Teo et al. (2022)
Neutrophil and erythrocyte	Neutrophil-erythrocyte hybrid membranes, Dexp-loaded hollow copper sulfide nanoparticles	OA induced by cutting off the anterior cruciate ligament	Mice/i.v.	IL-1*β*, TNF-*α*, IL-6 ↓	Xue et al. (2022)
BMSC	KGN, BMSC membrane-disguised Fe_3_ O _4_ nanoparticles	Articular cartilage damage induced by surgery	Rats/i.a.	Induced cartilage regeneration, more regenerated tissue with high quality of organized hierarchical architecture	Zhang, et al. (2021)

Abbreviations: ACAN, aggrecan; ADAMTS5, a disintegrin and metalloproteinase with thrombospondin 5; BMSC, bone marrow mesenchymal stem cell; COL2A1, collagen type II alpha 1; COL6A1, collagen type VI alpha 1; COX-2, cyclooxygenase-2; Dexp, *dexamethasone sodium phosphate;* KGN, Kartogenin; IL-8, interleukin-8; IL-17, interleukin-17; MSCs, mesenchymal stem cells; NGs, nano-ghosts; PGE2, prostaglandin E2; sGAG, sulfated glycosaminoglycan.

The cartilage regeneration is full of challenges in OA treatment. Stem cell therapy has been considered as a promising cartilage regeneration strategy in recent years. However, stem cell therapy has biosafety issues such as immune rejection and tumor transformation (Goldberg et al., 2017). Zhang et al. designed biomimetic stem cell membrane-disguised nanovehicles without biosafety risks for cartilage regeneration (Zhang et al., 2021). The Fe_3_O_4_ nanoparticles encapsulating Kartogenin (KGN) as the core of the biomimetic nanovehicles were disguised with natural bone marrow mesenchymal stem cell (BMSC) membrane. The KGN-loaded BMSC membrane-disguised Fe_3_O_4_ nanoparticles (KGN-MNPs) exhibits favorable biocompatibility and biosafety under the camouflage of cell membranes. KGN-MNPs enables rapid, high-quality cartilage regeneration in a cartilage defect rat model, suggesting a new, standardized strategy for future stem cell therapy. D'Atri et al. also developed a new type of biomimetic nanoparticles based on mesenchymal stem cells (MSCs), called nano-ghosts (NGs) (D'Atri et al., 2021). The NGs exhibited immunomodulatory capacity and were immune-evasive, while insensitive to host-induced changes. The researchers demonstrated that NGs can target cartilage tissue *in vitro* and *in vivo* and slow down the cartilage degeneration process. The results indicated that the NGs system might be a promising nanocarrier platform and potential immunomodulatory drug in the treatment of OA.

The pro-inflammatory phenotype (M1) and the anti-inflammatory phenotype (M2) macrophages in joints play important roles in OA. M2 macrophages can exhibit anti-inflammatory effects and present potential for treatments of OA (Fahy et al., 2014; Dai et al., 2018). Ma et al. constructed an artificial M2 macrophage (AM2M) with yolk-shell structure for disease-modifying OA therapy (Ma et al., 2021). The AM2M was prepared by using macrophage membrane as “shell” and inflammation-sensitive nanogel as “yolk”. The nanogel, which was fabricate by physical interaction of gelatin and chondroitin sulfate (ChS) burst release to suppress inflammation and repair the cartilage. In addition, the AM2M targeted to the inflamed area with long-term residence and blocked the immune stimulation induced by macrophages. The researchers revealed a promising therapeutic strategy to break a vicious and self-perpetuating cycle in OA patients. Teo et al. purposed a facile strategy to improve the efficacy of the macrophage membrane-derived nanoparticles (Teo et al., 2022). They prepared pro-inflammatory M1 and anti-inflammatory M2 macrophages through cell polarization, and then constructed M2 macrophage membrane-coated gold nanoparticles (Au-M2 NPs). In comparison with the nanoparticles generated from other macrophage subsets, the Au-M2 had the most effective pro-inflammatory cytokine sponge ability, and alleviated the inflammation and matrix degradation most significantly, in both IL-1*β* induced chondrocyte and the explant OA models. Au-M2 abolished IL-1*β*-induced MMP13 production and significantly suppressed nitric oxide production due to IL-1*β* stimulation. Compared to other therapeutic strategies based on cell membranes without special treatment, macrophage polarization may be a promising strategy for OA therapy.

Although neutrophil is the first responsive messenger to inflammation, presenting targeted ability to inflammatory sites with the CD11a ligand (Li et al., 2018), its application potential in cell-based nanotechnologies may be limited due to the short half-life (span of 7 h) (Kang et al., 2017; Chu et al., 2018). It has been reported that nanoparticles can obtain long-circulating delivery capacity after coated with erythrocyte membrane (Hu et al., 2011). Therefore, the neutrophil erythrocyte hybrid membranes camouflaged hollow copper sulfide nanoparticles (D-CuS@NR NPs) loading dexamethasone sodium phosphate (Dexp) were designed by Xue et al. for the treatment of OA (Xue et al., 2022). Under a NIR laser (1,064 nm), D-CuS@NR NPs exhibited excellent photothermal conversion capacity and controlled drug release, presenting good cytocompatibility and anti-inflammatory ability. It is noteworthy that, the hybrid biomimetic DCuS@NR NPs with NIR treatment can significantly reduce cartilage degeneration and alleviate synovial inflammation in the posttraumatic OA model.

### 2.4 Functional Nanotherapeutic Strategies for OA

Although many nanomaterial-based drugs and nanotherapeutic strategies have been investigated for OA therapy and many clinical studies on nanotherapeutic strategies are in progress (Dejulius et al., 2021; Hunter et al., 2022), the nanomedicines currently in clinical trials are mainly non-functional nano-scale/micron-scale materials loaded with old drugs that are already commercially available for OA therapy. They mainly extend the retention time of the drugs and reduce systemic diffusion (Wang et al., 2021). Some other functional nanotherapies for OA, with the extra properties of stimuli-responsive drug release, multiple regulatory mechanisms or other multifunctional characteristics, have attracted attention and are under investigation. These functional nanotherapeutic strategies are classified and summarized as shown in Table 3.

**TABLE 3:** Functional nanotherapeutic strategies for OA.

Category	Composition	Model	Animal/Delivery route	Outcome	Ref.
Photothermal-triggered	Photothermal-agents and NO molecules in nanoparticles	OA injected by papain solution	Mice/i.a.	TNF-*α*, IL-1*β*, IL-6, COX-2 and IL-8, P65, Notch-1 ↓, AMPK*α* ↑	Chen et al. (2019)
PH-responsive	Modified mesoporous silica nanoparticles (MSNs) with pH-responsive polyacrylic acid (PAA) for loading of Andrographolide(AG)	OA induced by transected anterior cruciate ligament	Rats/i.a.	MMP3, MMP13 ↓, COL2A1 ↑	He et al. (2021)
MMP-13 enzyme and pH- responsive	Poly (2-ethyl-2-oxazoline)-poly (PPL) with a specific peptide substrate of MMP-13 enzyme to form MR-PPL, psoralidin (PSO)	OA induced by injected of papain solution	Mice/i.a.	TNF-*α*, MMP-3 and MMP-13 ↓, COL2A1 ↑	Lan et al. (2020)
ROS-responsive	Tannic acid and crosslinker tetrahydroxydiboron	OA induced by injected MIA	Mice/i.a.	Arg1, IL-10 ↑, iNOS, IL-6, TNF-*α* ↓	Li X et al. (2021)
Thermoresponsive	HA conjugated to poly (N-isopropylacrylamide) (pNiPAM)	OA induced by surgery	Mice/i.a.	VEGF, IL-1*β*, TNF*α* ↓	Maudens et al. (2018b)
NIR laser- responsive	MPDA decorated MOF, rapamycin (Rap), bilirubin (Br)	OA induced treansected cruciate ligament	Rats/i.p.	ROS, TNF-*α*, IL-6, MMP9, ADAMTS5 ↓, Aggrecan, COL2A1 ↑	Xue et al. (2021)
Light-Responsive	Azobenzene-modified mesoporous silica nanoparticles (bMSNs-AZO) and *β*-cyclodextrin-modified poly(2-methacryloyloxyethylphosphorylcholine) (CD-PMPC)	—	Mice/i.a.	IL-6, MMP13, ADAMTS5 ↓; Agg ↑	Zhao et al. (2021)
Multifunctional: Oxidation-responsive, ROS scavenging, CO-releasing	CO donor (CORM-401), PDNs as the carrier, and FA-modified HA as the targeting ligand	OA induced by a single intra-articular injection of 5 mg Sodium iodoacetate (MIA)	Rats/local injection	HO-1 ↑, p38 MAPK, NF-kB (p50/p65), TLR-2, IL-1*β*, IL-6, TNF-*α* ↓	Yang G et al. (2020)
Biologically derived With multiple regulatory mechanisms	pBMSC secreted exosomes	OA induced by collagenase VII.	Mice/i.a.	Promote the proliferation and migration of chondrocytes. Acan, COL2A1 ↑	Zhou et al. (2020)

Abbreviations: Acan, aggrecan; Agg, the anabolic gene aggre-can; AMPK, Adenosine 5‘-monophosphate (AMP)-activated protein kinase; Arg1, Arginase-1; COL2A1, collagen type II; FA, folic acid; HA, hyaluronic acid; IL-6, interleukin-6; iNOS, inducible nitric oxide sunthase; MIA, monosodium iodoacetate; MMP13, matrix metalloproteinases-13; MOF, metal organic framework; MPDA, mesoporous polydopamine; NF-kB, nuclear factor-kappa B; NIR, near-infrared; NO, nitric oxide; OH-1, heme oxygenase-1; pBMSCs, polydactyly bone marrow-derived MSCs; PDNs, Peptide dendrimers nanogels; ROS, reactive oxygen species; TLR-2, toll like receptor 2; VEGF, vascular endothelial growth factor.

#### 2.4.1 Stimuli-Responsive NPs Based Therapeutic Strategies for OA

**FIGURE 5:**
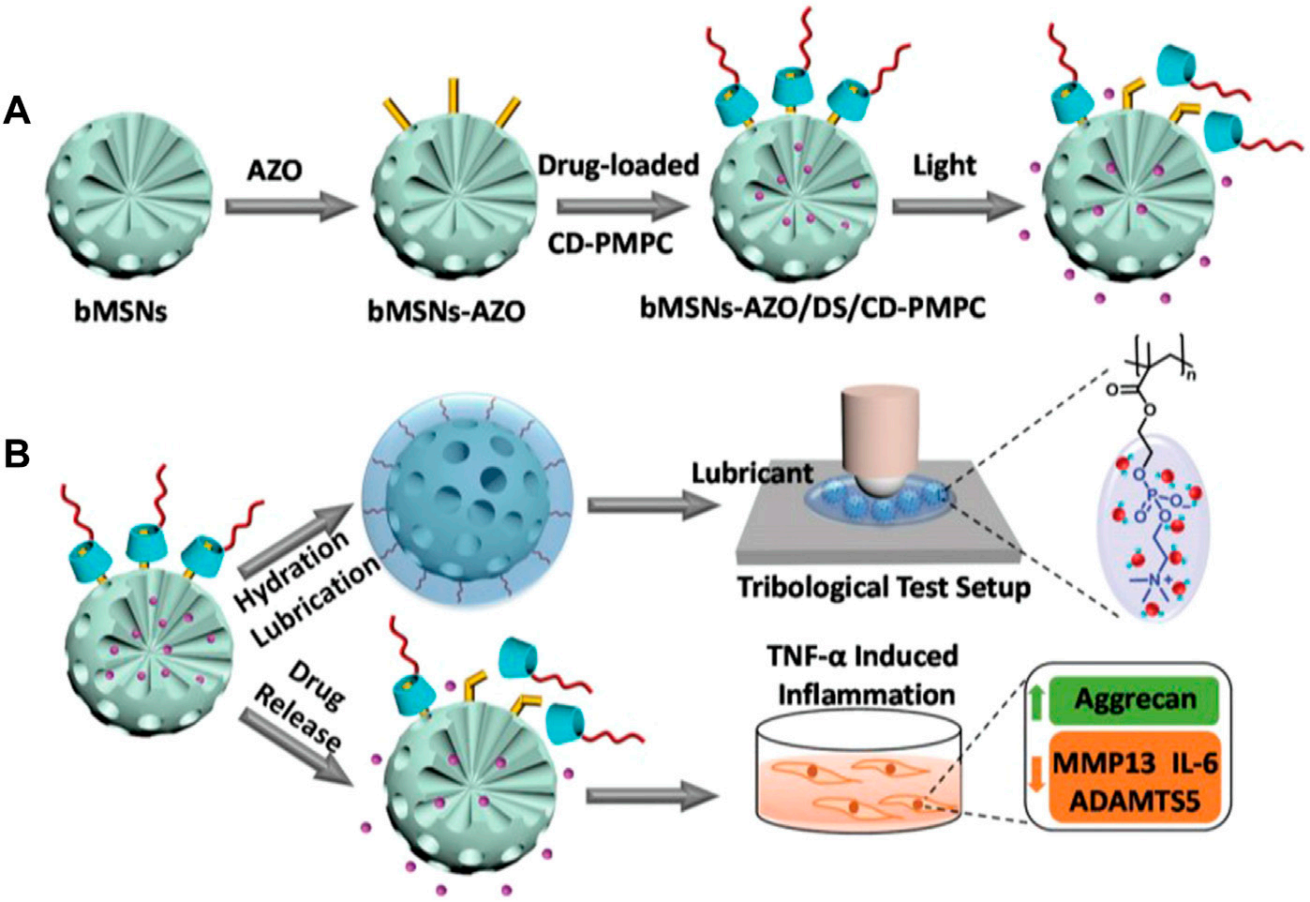
Drawing of the visible light-responsive dual-functional nanosystem with drug delivery and lubrication enhancement for OA. **(A)** Synthesis of bMSNs nanoparticles and the corresponding modification process. **(B)** Mechanism of lubrication enhancement and anti-inflammatory properties. Reproduced with permission (Zhao et al., 2021). Copyright 2021, Royal Society of Chemistry.

Stimuli-responsive NPs release their agents only when a suitable trigger or specific conditions are encountered. The disease-related local microenvironments, such as temperature, pH, and oxidative stress, or external stimuli, such as NIR light, provide design ideas and application opportunities for these types of nanocarriers with special functions (Lawson et al., 2021). Zhao et al. reported light-responsive dual-functional biodegradable mesoporous silica nanoparticles for the treatment of OA (Figure 5), which were constructed by supramolecular interactions between azobenzene-modified mesoporous silica nanoparticles (bMSNs-AZO) and *β*-cyclodextrin-modified poly(2-methacryloyloxyethyl phosphorylcholine) (CD-PMPC) (Zhao et al., 2021). The strategy combining light-responsive local drug release and lubrication enhancement is required for a synergistic effect on the treatment of OA (Moro et al., 2004). To achieve this effect, drug release can be induced after the nanoparticles pass through the dermal tissue by the isomerization of azobenzene, which can be triggered by visible light. Additionally, a photothermal-triggered nitric oxide nanogenerator combined with siRNA, NO-Hb@siRNA@PLGA-PEG (NHsPP), can suppress macrophage inflammation by efficiently converting absorbed NIR light energy into sufficient heat to trigger the production of NO (Chen et al., 2019). A cartilage-targeting peptide-modified dual-drug delivery nanoplatform with NIR laser response was constructed by Xue et al. for OA therapy (Xue et al., 2021). This dual-drug delivery system was based on the metal-organic framework (MOF)-decorated mesoporous polydopamine (MPDA) with rapamycin (Rap) loaded into the mesopores and bilirubin (Br) loaded onto the shell of the MOF,then, collagen II-targeting peptide (WYRGRL) was attached to the surface of the nanoplatform to prepare cartilage-targeting RB@MPMW. The system showed excellent ROS scavenging ability, high anti-apoptotic effects, enhanced autophagy activation, and chondrocyte protection, after the two agents being sequentially released *via* NIR laser irritation. RB@MPMW can promote chondrocyte mitochondrial energy metabolism in the inflammatory microenvironment, has a good MR imaging ability, which can monitor its therapeutic effects *in vivo*, and can significantly delay cartilage degeneration.

ROS are highly reactive molecules mainly generated by NADPH oxidase and mitochondria and play an important role in the physiological processes of OA. The functional nanoplatforms that respond to ROS for OA therapy have been investigated. The response mechanism of the ROS-responsive biomaterials can be divided into two types: degradation in response to ROS or changes in physicochemical properties (Yao et al., 2019). Li et al. prepared a boronate-stabilized polyphenol–poloxamer188 assembled dexamethasone nanodrug, which showed ROS-responsive drug-release behavior and ROS scavenging ability for macrophage repolarization for the treatment of OA (Li X. et al., 2021). This nanodrug efficiently inhibited ROS and nitric oxide production in lipopolysaccharide-activated RAW264.7 cells and modulated the polarization of macrophage M2 at a much lower concentration than free dexamethasone. Cartilage degradation and bone erosion in the joints were also inhibited by the nanodrug, along with the inhibition of proinflammatory cytokines.

The formation of lactate acidifies the microenvironment; the reduction of pH to as low as 6.0 was found in the articular cavity of OA patients (Goetzi et al., 1974). He et al. proposed a pH-responsive mesoporous silica nanoparticle-based drug delivery system with controlled release of andrographolide for treatment of OA (He et al., 2021). Modified MSNs with pH-responsive polyacrylic acid (PAA) were used for loading Andrographolide (AG) to form AG@MSNs-PAA. The nanocarriers had high drug-loading efficiency and pH-responsive properties, which were favorable for sustained release in the OA environment. AG@MSNs-PAA showed enhanced antiarthritic efficacy and chondroprotective capacity in chondrocytes stimulated by IL-1*β* and the anterior cruciate ligament transection-induced rat OA model.

Enzyme-responsive nanotherapies also permit precise control of the therapeutic effect only at the lesion of interest. The microenvironment of the hyaline cartilage was marked by matrix metalloproteinases-13 (MMP-13) overexpression and weak acidity in OA. (Meng et al., 2016). Lan et al. constructed a nano-micelle-based MMP-13 enzyme and a pH-responsive therapeutic nanoplatform for OA therapy (Lan et al., 2020). This nanoplatform was incorporated with a motif specifically targeting the cartilage, a motif responsive to MMP13 to specifically report OA condition and biodynamics of nanomicelles, psoralidin (PSO), and a biocompatible polymeric skeleton for sustainable drug release in response to OA. It promoted cell proliferation and inhibited inflammatory responses by down-regulating TNF-*α*, MMP-3, and MMP-13, and significantly alleviated the cartilage lesions. The high effectiveness of this functional nanotherapeutic strategy was confirmed by *in vitro* and *in vivo* studies.

**FIGURE 6:**
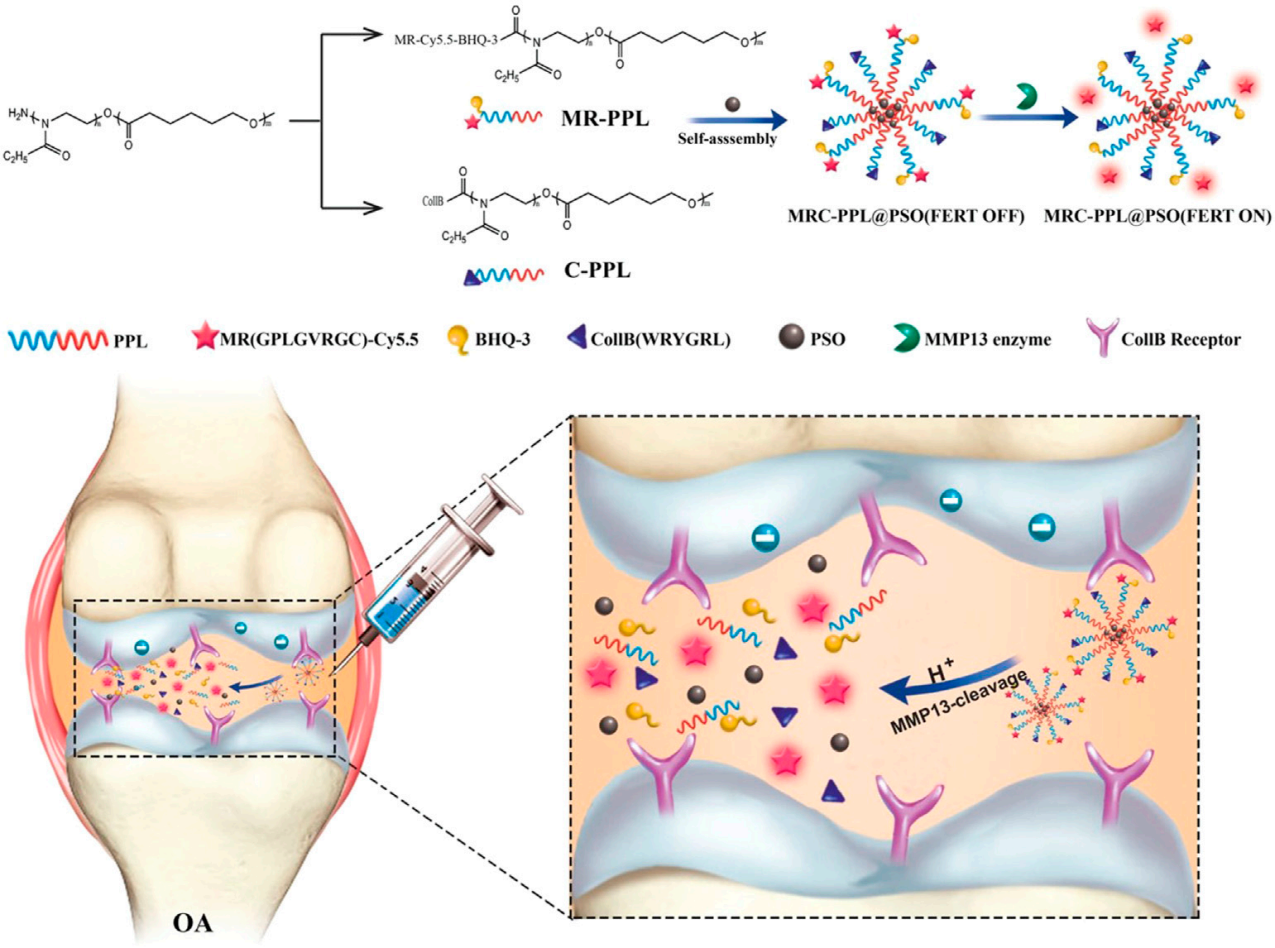
Strategy of MMP-13 and pH responsive theranostic nano-micelles for osteoarthritis. Reproduced with permission (Lan et al., 2020). Copyright 2020, BioMed Central Ltd.

Providing nanocarriers with a temperature-responsive function is also a promising strategy for the treatment of OA. The joints and the skin are often affected by an imbalance in the breakdown and production of hyaluronic acid (HA). HA alterations that confer thermosensitivity might help to prolong the *in vivo* lifetime/residence of HA. Therefore, the thermoresponsive poly (N-isopropylacrylamide) (pNiPAM) polymer might be suitable because of its well-defined sol-gel transition (Li Q. et al., 2021). Maudens et al. synthesized self-assembled thermoresponsive nanostructures of HA conjugates for OA therapy (Maudens et al., 2018b). This new injectable HA-pNiPAM was prepared by physically crosslinking hydrogel that could form nanoparticles spontaneously due to a change from room temperature to body temperature and was less sensitive to enzymatic degradation (Figure 6). This increased the residence time at the site of injection and improved joint treatment and dermatological applications. It was biocompatible and, thus, had a prolonged residence time at the site of injection, and also could protect the cartilage, suppress inflammation, and maintain the thickness of the epiphysis in patients undergoing OA treatment.

#### 2.4.2 Muti-Functional NPs Based Therapeutic Strategies for OA

Multi-functionalization of nanocarriers is a novel strategy to improve the efficacy of therapy by integrating different functions in one nanovehicle. For example, targeting efficiency, bioavailability, and biological activity can be improved by this method. The targeted and stimuli-responsive nanotherapies, and many other combinations of functions, have also been investigated for OA treatment (Wang et al., 2021). The specific inhibition of proliferation of activated macrophages and the clearance of high levels of ROS secreted by macrophages are important for the treatment of OA (Pu et al., 2014; Yang et al., 2019). CO is a peculiar endogenous signaling molecule with important cytoprotective effects and anti-inflammatory potential that does not cause multi-drug resistance and can prevent oxidative stress at the source. CO release molecules (CORMs) have different response release mechanisms (Schatzschneider, 2015), and the CO release rate of CORM-401 could be significantly increased in the presence of biologically relevant oxidants (e.g., H_2_O_2_) (Cai et al., 2016). Peptide-dendrimeric nanogels (PDNs) are a kind of polymeric material that have multiple functions and features (Fan et al., 2018). HA is also commonly injected intra-articularly for OA treatment, as it not only has good biocompatibility and biodegradability but also can prevent contact friction at the surface of the cartilage (Chen et al., 2014). Yang et al. examined novel multifunctional nanoparticles as anti-inflammatory drugs (CPHs) that were based on CO gas therapy (Yang G et al., 2020). The CPHs were constructed using CORM-401 as the CO donor, PDNs as the carrier, and FA-modified HA as the targeting ligand. Thus, CPHs had almost all the advantages of the constituent substances. They efficiently entered the activated macrophages through FA-mediated and HA-mediated specific targeting, and by depleting high levels of intracellular hydrogen peroxide, they rapidly released large amounts of CO intracellularly. The generated CO inhibited cell proliferation, induced the activation of heme oxygenase (HO-1), downregulated the expression of p38MAPK, NF-kB (p50/p65), and TLR-2, and also effectively inhibited the secretion of IL-1*β*, IL-6, and TNF-*α*. The *in vivo* results further demonstrated that CPHs can massively deplete ROS in OA-affected joints, effectively inhibiting the degradation of articular cartilage and its extracellular matrix. They are safe and do not show toxicity to normal macrophages.

Besides the abovementioned nanocarriers with responsive functions, nano-scale biologically derived vesicles with various regulatory mechanisms called exosomes also have multifunctional regulatory effects on the treatment of OA (Ni et al., 2019). Exosomes are a subtype of secreted vesicles that are released in the ECM when multivesicular bodies fuse with the cytoplasmic side of the plasma membrane. They are disc-shaped and have a diameter of 30–150 nm (van Niel et al., 2018). The pathophysiological functions of exosomes in OA have also been investigated in recent years. Mesenchymal stem cells (MSCs) are widely used for OA therapy, and exosomes might play a major role in the treatment (Bobis-Wozowicz et al., 2015). Zhou et al. reported a special kind of exosome secreted by bone marrow-derived mesenchymal stem cells (BMSCs) from congenital polydactyly tissue. These exosomes could alleviate osteoarthritis by promoting chondrocyte proliferation (Zhou et al., 2020). The results showed that the polydactyly bone marrow-derived MSCs (pBMSCs) had a greater ability than the BMSCs to differentiate into chondrocytes, and the migration and proliferation of chondrocytes were stimulated by exosomes secreted by pBMSC (pBMSC-EXOs). Additionally, injecting pBMSC-EXOs and the exosomes secreted by BMSC (BMSC-EXOs) attenuated OA in an OA mouse model. The pBMSC-EXOs had a stronger therapeutic effect than the BMSC-EXOs. They improved cartilage injury in the knee of a collagenase-induced OA mouse model, evaluated based on the OARSI score. The study showed that pBMSC-EXOs, which were once considered “medical waste”, can be used for the treatment of OA when administered properly.

### 2.5 Other Nanotechnology-Based Synergistic Therapeutic Strategies

Gold (Au) compounds are effective for the treatment of various inflammatory diseases, but the use of Au compounds is limited due to their side effects. The safety evaluation of gold nanoparticles (AuNPs) in osteoarthritis (OA) treatment is vague. Abdel-Aziz et al. biosynthesized, characterized, and evaluated the effects of AuNPs and/or Diacerein® (DIA) for OA treatment. (Abdel-Aziz et al., 2021). The synthesized AuNPs and DIA significantly reduced serum inflammatory cytokines, improved biochemical parameters, estrogen levels, and cartilage joint histology of OA rats. They demonstrated that the AuNPs were more effective than DIA, and the combined therapeutic strategy presented more effectively than the isolated, which indicated that AuNPs are promising nanomaterials for OA therapy, both alone and in combination with DIA.

Studies have shown that ROS and reactive nitrogen species (RNS) both participate in OA development and progression significantly. Hence, antioxidants might be effective in osteoarthritis (OA) therapy (Mazzetti et al., 2001). Some antioxidants, such as melatonin and N-acetylcysteine (NAC), were investigated and found to be retained in the joint for a short while only (Schreck et al., 1991; Lim et al., 2012). Natural melanin protects the skin from ultraviolet (UV) irradiation due to its surface quinone residues. The antioxidant mechanisms of artificial melanin nanoparticles were investigated and the results showed that these materials can scavenge many types of radicals (Liu et al., 2013; Liu et al., 2017). Zhong et al. investigated dopamine melanin (DM) nanoparticles to scavenge ROS and RNS for OA therapy (Zhong et al., 2019). These DM nanoparticles retained at the injection site after intra-articularly injection, presenting a strong ability to sequester ROS and RNS with slight cytotoxicity. The DM nanoparticles exhibited anti-inflammatory and chondroprotective effects on IL-1*β*-induced chondrocytes. When investigated in an OA model *in vivo*, these nanoparticles decreased the release of inflammatory cytokines and minimized the loss of proteoglycans, which alleviated cartilage degradation. Further mechanistic studies showed that DM nanoparticles considerably elevated the expression levels of autophagy markers in IL-1*β*-stimulated chondrocytes and stimulated autophagy for chondrocyte protection, which helped to control OA (Mi et al., 2018).

## 3 Summary and Perspective

With the growth of the global aging population and the increase in the proportion of obese people, the incidence of OA is expected to increase, the resulting disability rate, productivity loss, and medical economic burden need to be effectively prevented. The complexity of the pathogenesis of OA has limited the efficacy of traditional single therapies and led to the failure of a series of anti-cytokine therapies in clinical trials. Currently, most of the clinical treatment strategies for OA focus on delaying the development of the disease, reducing pain, and improving motor functions. However, it is difficult to achieve an optimal therapeutic effect through traditional clinical treatment strategies due to the complexity of molecular and cellular alterations in the cartilage tissue of OA patients, the effective treatment of osteoarthritis has many obstacles and lacks a suitable cure (Van Spil et al., 2019; Mao et al., 2021).

Due to the continuous advancements in nanotechnology and the growing understanding of the pathological mechanisms of OA, different nanoparticles have been widely explored in the therapy of OA and other diseases. The main benefits of these drug delivery systems based on different nanoparticles are that they can release drugs over a long time, increase the retention of drugs in the joints, and enhance therapeutic efficacy due to functional regulatory strategies. Thus, using these systems can lower the therapeutic dose, reduce the frequency of administration, increase pharmacological efficacy, and reduce off-target toxicity. Besides these abovementioned strategies, novel fields, such as chondroprotective treatment (Yang J et al., 2020), nanomaterial-based scaffolds for cartilage regeneration (Arslan et al., 2018; Mahboudi et al., 2018), and other strategies of immunotherapy, are being developed. Moreover, changes in the meniscus are often occur in the progression of OA (Katsuragawa et al., 2010), and the repair and regeneration of meniscus injury is of great significance for preventing the progression of OA. Some novel tissue engineering technologies have also attracted extensive attention and evaluation recently (Murphy et al., 2019), and it is also promising to develop novel nanomaterial-based tissue engineering thechnologies for meniscal repair and regeneration in OA treatment.

However, the field of nanotherapeutics related to the clinical treatment of OA is still in its infancy. Many of the available nanocarriers have limitations, as the drugs need to be loaded for osteoarthritis treatment, and sustained-release systems in clinical use are uncommon (Jiang et al., 2018). Besides, the construction of functional nanoparticles also remains challenging. Synthesized nanomaterials are susceptible to a large number of proteins expressed on various cell membranes after entering the body (Thanuja et al., 2018). In order to add more functions to nanoparticles and enhance their efficacy, some polymer materials are often used to functionalize the surface of nanoparticles. But due to the ideal biocompatibility and antigenicity, these polymeric materials often trigger immune reaction and form the “protein corona” (Corbo et al., 2017). Natural cell membrane-modified biomimetic nanocarriers have better potential for clinical application as highly biocompatible materials, and have also demonstrated excellent therapeutic effects in the treatment of OA. However, the preparation of cell membranes currently requires cell lysis and cell membrane purification, it may cause contamination of the product and damage to the key proteins. Furthermore, due to the limited advantages of cell membranes derived from single cells, many researchers have developed and investigated novel nanotherapeutic strategies based on hybrid cell membranes (Xue et al., 2022). Moreover, owing to the rapid development and continuously optimization of nanomaterials, synthetic techniques and relative ligands, much more common issues may be addressed, including nanocarrier stability, satisfactory retention time, slight side effects in non-target tissues, and systemic biosafety. The potential of nanotherapeutic strategies for OA shall increase in the future.
